# Dual‐Targeted Trimyristin Nanoemulgel Accelerates Burn Wound Healing via VEGFR and FGFR Modulation

**DOI:** 10.1002/cbdv.71873

**Published:** 2026-10-22

**Authors:** Ankita Sinha, Parag Jain, Alka Patel, Kushagra Nagori, Ajazuddin

**Affiliations:** ^1^ Department of Pharmacology Rungta College of Pharmaceutical Science and Research Bhilai Chhattisgarh India; ^2^ School of Pharmacy Rungta International Skills University Bhilai Chhattisgarh India; ^3^ Department of Medicinal Chemistry Rungta College of Pharmaceutical Science and Research Bhilai Chhattisgarh India; ^4^ Department of Pharmaceutics Rungta College of Pharmaceutical Science and Research Bhilai Chhattisgarh India

**Keywords:** burn wound healing, FGFR, lipid nanocarrier, molecular docking, nanoemulgel, topical drug delivery, Trimyristin, VEGFR

## Abstract

Burn injuries remain a major therapeutic challenge due to delayed healing, infection, and poor tissue regeneration. This study aimed to develop and evaluate a trimyristin‐loaded nanoemulgel for enhanced healing of second‐degree burn wounds. A trimyristin nanoemulsion was prepared by high‐energy emulsification and incorporated into a Carbopol‐based gel matrix. The formulation exhibited excellent physicochemical stability, sustained drug release, and favorable skin compatibility. Molecular docking demonstrated strong interactions of trimyristin with Vascular Endothelial Growth Factor Receptor (VEGFR) and Fibroblast Growth Factor Receptor (FGFR), with binding affinities of −11.74 and −10.21 kcal/mol, respectively. In vivo studies in Wistar rats showed that the high‐dose nanoemulgel achieved 96.2% wound contraction by day 14 and reduced epithelialization time to 11.3 ± 0.6 days compared to 15.8 ± 0.9 days in controls. Histopathological analysis confirmed near‐complete tissue regeneration, highlighting the potential of trimyristin nanoemulgel for advanced burn wound therapy.

## Graphical abstract

The graphical abstract encapsulates the formulation of a trimyristin‐loaded O/W nanoemulgel and its dual‐targeted mechanism for burn wound healing. Trimyristin had significant affinity for VEGFR and FGFR, promoting angiogenesis, collagen production, and re‐epithelialization, while inhibiting inflammation and oxidative stress, so expediting tissue regeneration and wound healing.

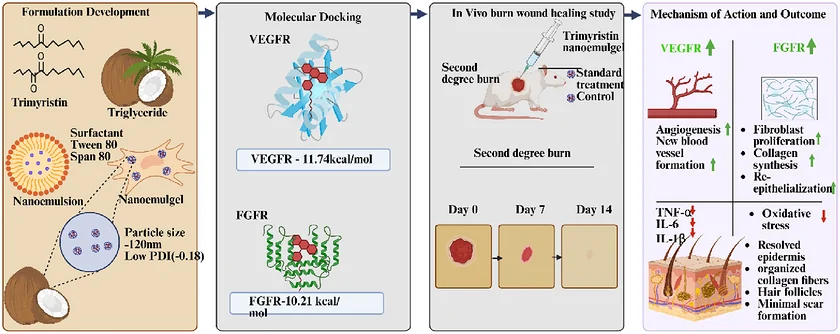

## 1 Introduction

Second‐degree burns or partial‐thickness burns involve the complete epidermis plus a portion of the dermis, either the outermost squamous cell layer or deeper reticular layer, differentiating them from full‐thickness injuries that penetrate beyond the skin. These burns generally manifest as painful and sensitive lesions, frequently accompanied by blisters, erythema, or mottled skin, a wet or exudative appearance, and pronounced oedema. Pain often gets worse when exposed to air or changes in temperature [1]. A comprehensive global health report estimated over 8.4 million new burn cases in 2019, leading to over 11,1000 fatalities; the majority of the burden is concentrated in low‐ and middle‐income countries, where hazardous cooking methods and restricted access to acute care are prevalent [2]. The diagnostic constraints, coupled with a significant risk of infection and protracted scarring, highlight the pressing necessity for sophisticated topical treatments that ensure targeted, prolonged distribution of anti‐inflammatory, antioxidant, and antibacterial compounds [3].

A burn injury triggers the generation of early reactivity of oxygen species, pro‐inflammatory cytokines (including TNF‐α, IL‐6 and IL‐1β), and a persistent influx of neutrophils and M1 macrophages, as well as which can affect the healing process if improperly regulated. Studies on the pathophysiology of burns have shown that cytokine overexpression may continue from days 3 to 7 post‐burn in conditions of persistent oedema or infection, highlighting the reduction of hyperinflammation as a crucial therapeutic target. Docking‐based ligand selection is ideal for targeting molecular regulators such as NF‐κB, Nrf2/ARE, PI3K/Akt, and MMP1/9 [4].

In silico docking facilitates the targeted identification of phytochemical ligands with beneficial binding affinities to burn‐related targets (e.g., NF‐κB subunits, MMP‐9, TNF‐α receptors, PI3K/Akt‐binding domains). Multiple studies indicate the effective binding affinities of trimyristin to various targets, with anticipated hydrogen bond interactions corresponding to the reported in vivo efficacy (e.g., decreased IL‐6 expression, increased HO‐1 induction). The docking results function as preclinical filters for incorporating drugs into trimyristin‐based nanoemulgel systems to optimise molecular targeting of burn lesions [5].

Trimyristin is a saturated triglyceride composed of three myristic acid molecules and glycerol. It is a solid which varies in colour from white to yellowish‐grey, with a melting point of around 56°C–57°C. It is abundant in nutmeg and is often isolated in organic chemistry laboratories due to its high yield and crystallinity. In nanotechnology and pharmaceuticals, it serves as a solid lipid matrix to create liquid nanoparticles and nanoemulsions based on trimyristin, guaranteeing superior encapsulating effectiveness, controlled release, and physical stability [6].

Nanoemulgels, hydrophilic polymer gels containing extremely small oil‐in‐water emulsions, offer a novel medium for the synergistic administration of pharmaceuticals in the management of second‐degree burns. This nanoemulsion component enhances the solubility and cutaneous absorption of the lipophilic actives, promoting efficient permeation into the stratum corneum [7]. The hydrogel polymer simultaneously increases viscosity and retention time, maintains a moist wound environment, and diminishes trans‐epidermal water loss, thereby reducing irritation and enhancing topical adhesion and patient compliance. These features enable regulated, prolonged release, thermodynamic durability, and increased local bioavailability of incorporated agents, leading to expedited re‐epithelialization, reduced inflammation, and greater collagen deposition in burns and cutaneous wounds [8].

## 2 Materials and Methods

### 2.1 Materials

Materials such as trimyristin, Tween 80, Span‐80, castor oil, triethanolamine, ethanol as solvent, KBr, and double‐distilled water were obtained from the Rungta College of Pharmaceutical Sciences and Research in Bhilai.

### 2.2 Molecular Docking Protocol

A molecular docking study was performed to assess the binding affinity and interaction characteristics of trimyristin with essential receptors involved in wound healing, specifically Endothelial Growth Factor Receptor (EGFR), Vascular Endothelial Growth Factor Receptor (VEGFR), TGF‐βR, FGFR, and PDGFR. Receptor structures were retrieved from the Protein Data Bank (PDB) using their respective IDs: 7KPB (EGFR), 8HBM (VEGFR), 1UHL (TGF‐βR), 2AZ5 (FGFR), and 2ACL (PDGFR). Trimyristin's ligand structure has been developed using standard methods and energy minimization to provide it an ideal form for docking [9]. Molecular docking simulations were performed using a validated software suite, with each receptor‐ligand pair subjected to grid‐based docking techniques under default parameters. Binding energies were recorded in kcal/mol, and docking run durations were noted to assess computational efficiency. The post‐docking analysis involved a comprehensive examination of hydrogen bonding and hydrophobic connections between trimyristin and receptor residues, highlighting essential interactions such as ARG419 and LYS558 in EGFR, ARG322 and GLN349 in VEGFR, and TYR215 and GLU253 in TGF‐βR. The interaction maps were visualized and annotated to identify potential hotspots for therapeutic modulation. All receptor structures were referenced via their respective PDB DOIs to ensure reproducibility and structural validation [10].

### 2.3 Characterization of Trimyristin

#### 2.3.1 FTIR Analysis

The functional groups of trimyristin were discovered by Fourier‐transform infrared (FTIR) spectroscopy. In a 1:100 (w/w) ratio, a small amount of the finely crushed pure trimyristin sample was mixed with dry potassium bromide (KBr). The pressure of a hydraulic press was then utilised to compact the mixture into a transparent pellet. Using an FTIR spectrophotometer, the pellet's FTIR spectra were captured between 4000 and 400 cm^−^^1^. The occurrence of ester and fatty acid groups has been confirmed by analysing and comparing characteristic absorption peaks with standard parameters [11].

#### 2.3.2 Solubility

Trimyristin's solubility was qualitatively determined with different solvents at ambient temperature. Approximately two millilitres of each solvent (hexane, ethanol, acetone, chloroform, and water) were combined with roughly 10 mg of trimyristin in different test tubes. The components were violently mixed before being allowed to stand for 10 min. Based on the degree of dissolution, the solubility was visually assessed and categorised as soluble, partially soluble, or insoluble. Trimyristin's polarity and solubility in various solvents were determined using the recorded outcomes [12].

### 2.4 Preparation of Trimyristin Nanoemulsion

The preparation of a trimyristin‐loaded oil‐in‐water (O/W) nanoemulsion was achieved through a combination of low‐energy spontaneous emulsification and moderately high‐energy approaches to ensure stability and nanoscale droplet dimensions. The oil phase was generated by dispersing trimyristin (300 mg or 500 mg per 50 mL formulation batch) in an appropriate co‐solvent, such as ethanol, to enhance the solubility and uniform distribution of the drug. This solution was incorporated with castor oil and a surfactant blend of Tween 80 (hydrophilic) and Span 80 (lipophilic) in a modified ratio to achieve a hydrophilic‐lipophilic balance (HLB) of 10–12, optimal for generating O/W emulsions. The entire oil phase was then heated to around 60 °C to ensure complete dissolve and reduce viscosity. The aqueous phase comprising 22 mL of distilled water was heated to 60°C. The warm oil phase was incrementally introduced into the aqueous phase while sustaining continuous electromagnetic stirring at 1000–1500 rpm for 15 min to form a coarse pre‐emulsion. The optimized surfactant blend (Tween 80 and Span 80) produced an HLB value of approximately 10–12, favoring the formation of an oil‐in‐water (O/W) nanoemulsion. The aqueous phase served as the continuous external phase, confirming the O/W nature of the system. The mixture underwent sonication at 5000 rpm for 15 min to diminish droplet size to the nanometre scale, followed by homogenized at 1500–3000 rpm to enhance stability and decrease droplet aggregation. The final formulation resulted in a transparent to clear nanoemulsion with uniformly distributed trimyristin droplets, exhibiting enhanced kinetic stability and their suitability for therapeutic applications [13].

### 2.5 Characterization of Nanoemulsion

#### 2.5.1 Particle Size Distribution and Polydispersity (DLS)

The average size of the drops and the polydispersity index (PDI) of the nanoemulsions were measured at 25°C using a dynamic light scattering method with the Zeta sizer Nano‐S laser nanoparticle dimension analyser. Every test was carried out three times. Distilled water was used to dilute the NE (1/100). The size distribution of the nanodroplets was then determined [14].

#### 2.5.2 Zeta Potential Measurement

The stability of nanoemulsion droplets and their zeta potential were predicted using dynamic light scattering. The sample was initially diluted to achieve moderate conductivity with pH modification as a possible alternative based on the formulation. The folded‐capillary zeta vessel was carefully filled with the diluted emulsion, preventing air bubbles and maintaining a clean contact with the electrodes [15]. The cell was enabled to stabilise in the instrument at roughly 25 °C for 2‐5 min. Measurements were taken over 10 successive runs utilising an applied voltage of approximately 60 V with automated polarity adjustment. The device measured electrophoretic mobility, which was subsequently utilised to determine the zeta potential via the Henry equation. The results were expressed as mean ± standard deviation, and values over ±30 mV were regarded as indicating high electrostatic stability and resistance to droplet aggregation [16].

### 2.6 Preparation of Hydrogel

Carboxymethyl cellulose (5% w/w) was gradually added to the distilled water while being gently agitated (300–600 rpm) to produce a smooth, lump‐free dispersion. The substance was allowed to hydrate at room temperature for two to three hours in order to ensure full swelling. Triethanolamine (TEA1.0% w/w) was incorporated after hydration to help change the gel's viscosity and substantially maintain the formulation, and pH was adjusted to 6.5–7.0. The produced gel was stored at ambient temperature in air‐tight containers after air bubbles were eliminated using vacuum or mild sonication [17].

### 2.7 Incorporation of Nanoemulsion into Hydrogel (Nanoemulgel Formation)

The nanoemulsion was carefully incorporated into the hydrogel base to maintain structural integrity while producing a homogeneous final composition. trimyristin (300 mg or 500 mg per 50 mL formulation batch) of the produced nanoemulsion was slowly added to the hydrogel base while gently and continuously stirring (100‐300 rpm) until the texture was optically uniform, ensuring that the droplets of the nanoemulsion were dispersed uniformly throughout the hydrogel matrix. To retain the required viscosity and spreadability, double‐distilled water was added to get the final volume approximately 50 mL [18].

### 2.8 Characterization of Nanoemulgel

#### 2.8.1 Rheology and Viscosity Measurements

The rheological properties and viscosity of the formulated nanoemulgel were assessed utilizing a Brookfield viscometer equipped with an appropriate spindle (often spindle no. 64) at ambient temperature (25°C ± 1°C). Approximately 10 g of the nanoemulgel formulation sample was immersed in a beaker and exposed to rotational shear at varied RPMs (usually 10, 20, 50, and 100 rpm) to assess flow behaviors. Measurements were measured in centipoise (cP), and rheological profiles were presented as viscosity against shear rate to evaluate pseudoplastic or thixotropic characteristics [19].

#### 2.8.2 Melting Point

The degree of melting of the nanoemulgel was obtained by utilising an automatic melting point device. A small quantity of dried sample was positioned in a capillary tube and subsequently introduced into the melting point device. The temperature was progressively elevated at a predetermined rate of 1°C–2°C per minute, and the measurement of the temperature at which the initial drop of liquid material emerged was determined as the melting point [20].

#### 2.8.3 PH

The pH level of an emulgel is determined using an electronic pH meter standardised with standard buffer solutions. A specific quantity of the preparation has been diluted with distilled water. And allowed to rest for two hours before testing. The glass electrode is completely immersed in an emulgel, and pH measurements must be recorded in three separate batches. Skin‐compatible emulgels generally possess a pH between 5.8 and 6.5, with a target of approximately 6.0 to reduce irritation [21].

#### 2.8.4 Spreadability Evaluation

The Nanoemulgel formulation's spreadability was assessed using a laboratory‐modified device intended for this study. It includes a wooden block, which is affixed to a pulley at one end. A rectangular ground glass plate of 20 cm by 20 cm was affixed to the block. 2 g of the formulation were positioned between the stationary ground plates and a separate glass plate of identical dimensions to the fixed ground plate. The movable glass panel is equipped with a hook. A 300 g weight was positioned at the apex of both plates for five minutes to eliminate air and ensure a consistent film of the formulation between the plates. The excess formulation was removed from the edges. The upper plate was subsequently placed underneath a force of 30 g, originally facilitated by a string connected to the hook and crossing over the pulley. The duration taken by the top plate to cover a distance of 10 cm was recorded. A reduced time signifies enhanced spreadability [22].

### 2.9 In Vivo Burn‐Wound Evaluation

#### 2.9.1 Ethical Approval (IAEC/CPCSEA)

All protocols involving animals were reviewed and approved by the Institutional Animal Ethics Committee (IAEC) in accordance with the rules set out by the Government of India's Committee for the Purpose of Control and Supervision of Experiments on Animals (CPCSEA), according to registration number RCPSR/2024/IAEC/24.

#### 2.9.2 Wistar Rat Model and Burn Induction

The Wistar rats were randomly selected around 250 g to ensure that there were no underlying systemic illnesses, infections, or skin abnormalities. Before the procedure, the dorsal surfaces of the rats were shaved and cleaned with sterile normal saline. Ketamine hydrochloride was given as the anaesthetic agent to ensure sleepiness and pain management. Burn wounds were made using a cylindrical metal rod that had been preheated in boiling water or a heating block equipped with a thermostat. The rod was employed for a few seconds to produce a partial‐thickness burn on the shaved dorsal region without puncturing the underlying muscle tissue. The region of the wound was then cleaned with sterile gauze. The animals were subsequently taken back to their cages for observation [23].

#### 2.9.3 Group Allocation and Treatment Regimens

The animals were substantially selected to five groups (*n* = 4 per group) to reduce selection bias following burn induction. The control group had burn wounds that remained untreated to evaluate the natural healing process, whereas the diseased group had burn wounds induced under conditions mimicking impaired healing to serve as a pathological model. The standard group received a commercially available wound‐healing ointment as the reference treatment. Treatment Group 1 (T1) was administered a nanoemulsion‐loaded hydrogel containing Trimyristin at a concentration of 300 mg/50 mL, while Treatment Group 2 (T2) received the same formulation at a higher concentration of 500 mg/50 mL. All treatments were applied topically once daily for 14 consecutive days under aseptic conditions. Before application, the wound surface was gently cleansed with sterile normal saline to promote optimal absorption and minimize the risk of contamination [24].

#### 2.9.4 Wound Healing Assessment—Rate of Contraction and Period of Epithelialization

The measure of the percentage of contraction of the wound, the degree of wound closure, and the duration of epithelial formation were utilized to assess the wound healing area. The subsequent formula was employed to calculate the percentage of contracted wounds, considering that the initial wound size was 100% [25].

$$\begin{array}{ccc} & & \%\mathrm{wound}\mathrm{contraction}\\ & & =\left(\mathrm{Initial}\mathrm{wound}\mathrm{area}-\mathrm{Specific}\mathrm{day}\mathrm{wound}\mathrm{area}\right)\\ & & /\mathrm{Initial}\mathrm{day}\mathrm{wound}\mathrm{area}\times 100 \end{array}$$

#### 2.9.5 Histopathological Examination

Animals were put under anaesthesia and placed on the surgical table on days 0 and 14 after lesion induction. The healthy skin surrounding the wound, including the fascia muscles, was removed. Haematoxylin‐eosin (H&E) staining was used after the specimens were immersed in paraffin, fixed with 10% neutral buffered formalin, and cut into 4 µm thick slices using a microtome cutter. A researcher evaluated the specimens' healing status and the degree of histopathologic modifications using light microscopy. Re‐epithelialization, granulation tissue, pro‐inflammatory cells, and angiogenesis are the four histological indicators that were used to score wound healing using the Hazrati method. Individual criterion scores were added to determine the overall healing score in each event; lower values denoted worse wound healing [26].

### 2.10 Statistical Analysis

Each value in the data is represented as the mean ± standard deviation (SD). Statistical analysis was performed by utilizing GraphPad Prism 8.0. Wound contraction data were evaluated with two‐way ANOVA, followed by intergroup comparisons at each time point. Data from multiple groups were evaluated with one‐way ANOVA. *P* < 0.05 was the acceptable value for statistical significance [27].

## 3 Results

### 3.1 Characterization of Trimyristin

#### 3.1.1 FTIR Analysis of Trimyristin

The FTIR spectrum of trimyristin displayed unique peaks indicative of its distinctive functional groups and molecular structure. The absorption bands at 2918.80 and 2852.76 cm^−^^1^ correspond to C–H stretching vibrations in aliphatic –CH_2_ and –CH_3_ groups, signifying the existence of extended alkyl chains [28]. A prominent peak at 1735.77 cm^−^^1^ corresponds to the stretching vibration of the ester carbonyl (C═O), so affirming the existence of the ester functional group (Figure 1). The interval from 1267.73 to 1106.93 cm^−^^1^ exhibits significant C–O stretching vibrations, hence reinforcing the presence of the ester bond. The peaks at 1468.73 and 1398.38 cm^−^^1^ correspond to CH_2_ bending vibrations, indicative of methylene groups present in fatty acid chains. The signal at 719.29 cm^−^^1^ indicates CH_2_ rocking modes, hence confirming the existence of long hydrocarbon chains [29].

**FIGURE 1:**
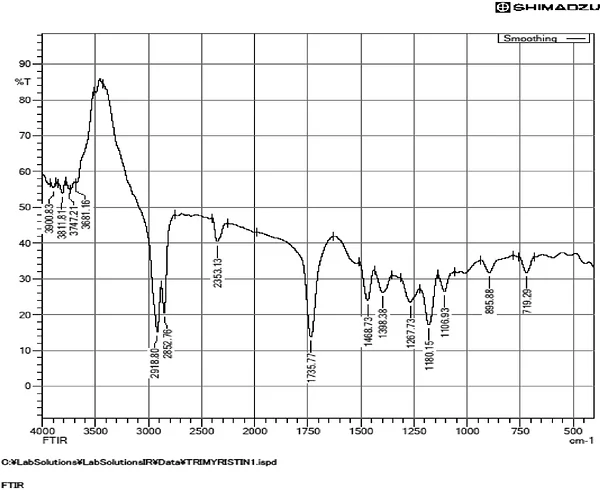
FTIR spectrum for Trimyristin.

#### 3.1.2 Solubility of Trimyristin

Trimyristin's hydrophobic nature made it extremely insoluble in water, but it dissolves well in organic solvents like hexane and chloroform. Ethanol, ethyl acetate, and diethyl ether exhibited moderate solubility, while polar solvents such as methanol, propylene glycol, and DMSO displayed variable or reduced solubility.

### 3.2 Nanoemulsion Characteristics

#### 3.2.1 Particle Size Distribution and Polydispersity

The produced trimyristin nanoemulsions were shown to have a low polydispersity index (PDI) of 0.18 ± 0.02 and a narrow size distribution, with an average droplet size of 120 ± 10 nm, according to a dynamic light scattering study. The small droplet size and low PDI indicate the production of a homogenous nanoscale emulsion, which is beneficial for increased stability and bioavailability.

#### 3.2.2 Zeta Potential

The zeta potential of the nanoemulsion formulations was measured to evaluate their surface charge and colloidal stability using a dynamic light scattering‐based zeta potential analyzer (e.g., Malvern Zetasizer Nano ZS). Prior to analysis, samples were diluted tenfold with deionized water to minimize multiple scattering and maintain appropriate conductivity. Measurements were conducted at 25°C ± 1 °C using gold‐plated disposable capillary cells, with the pH adjusted to physiological range (6.5–7.4) when necessary. Electrophoretic mobility was recorded and converted to zeta potential values using the Smoluchowski approximation, assuming spherical particle geometry in aqueous medium. Each sample was analyzed in triplicate, and the results were expressed as mean ± standard deviation. A zeta potential magnitude exceeding ±30 mV was considered indicative of good electrostatic stability, suggesting minimal aggregation and favorable dispersion characteristics of the nanoemulsion system. The trimyristin nanoemulsion exhibited zeta potential values of −32.5 ± 1.8 mV (300 mg/50 mL) and −35.8 ± 2.1 mV (500 mg/50 mL), indicating excellent colloidal stability and low aggregation tendency.

### 3.3 Nanoemulgel Properties

#### 3.3.1 Viscosity

Viscosity evaluation showed that the nanoemulsion hydrogel with 500 mg/50mL of Trimyristin had a significantly greater viscosity (5700 cP) than the 300 mg/50 mL formulation (4400 cP). This increase is due to the greater lipid content, which expands the internal phase volume and increases flow resistance. The data clearly show that drug concentration greatly changes the gel's viscosity, which can affect application and drug release.

#### 3.3.2 Melting Point

The melting point was 54.5°C ± 0.5°C for the formulation containing 300 mg/50 mL of trimyristin and 55.3°C ± 0.4°C for the formulation containing 500 mg/50 mL of trimyristin. The partial amorphization caused by nanodispersion and interaction with formulation components is shown by the fact that both melting points are marginally lower than the melting range of pure trimyristin (56°C–57°C). These melting points facilitate drug release during application while ensuring the gels remain stable at normal skin temperatures.

#### 3.3.3 PH

The pH of nanoemulgel formulations containing distinct Trimyristin concentrations was evaluated to determine their skin compatibility and to ensure minimal irritation when applied topically. The formulation containing 300 mg of trimyristin had a pH of 6.7 ± 0.1, whereas the formulation containing 500 mg/50 mL of trimyristin had a slightly higher pH of 6.9 ± 0.1. Those two values are within the optimal pH range for skin, which is approximately 5.5 to 7.0; thus, the formulations can be administered topically without harming the skin's acidic layer.

#### 3.3.4 Spreadability

The nanoemulgel compositions' spreadability was investigated to determine their uniform skin covering and application simplicity. The formulation containing 300 mg/50 mL of trimyristin exhibited favourable spreading properties, achieving a spread diameter of 7.8 ± 0.2 cm. The 500 mg/50mL Trimyristin formulation exhibited diminished spreadability, measuring 7.2 ± 0.3 cm in diameter, attributable to its elevated viscosity. Both formulations exhibited sufficient spreadability values for topical application, promising patient adherence and effective use (Table 1).

**TABLE 1:** Physicochemical Properties of Trimyristin Nanoemulgel Formulation**s**.

Parameter	300 mg/50 mL Trimyristin	500 mg/50 mL Trimyristin	Interpretation
Viscosity (cP)	4400	5700	↑ Lipid content increases internal phase volume and flow resistance
Melting point (°C)	54.5 ± 0.5	55.3 ± 0.4	Slightly lower than pure trimyristin (56–57°C); indicates partial amorphisation
pH	6.7 ± 0.1	6.9 ± 0.1	Within skin‐compatible range (5.5–7.0); safe for topical application
Spreadability (cm)	7.8 ± 0.2	7.2 ± 0.3	Higher viscosity reduces spread diameter; both suitable for uniform application

### 3.4 Molecular Docking Outcomes

The molecular docking investigation of Trimyristin with major receptors implicated in wound healing demonstrates favourable interactions that highlight its medicinal potential. The most favourable binding affinity among the five receptors under investigation was demonstrated by VEGFR (PDB ID: 8HBM), which showed robust molecular engagement with a docking energy of −11.7434 kcal/mol. Trimyristin established several hydrogen bonds with residues including ARG322, TYR330, and GLN349, indicating strong stabilisation within the active site. FGFR (2AZ5) exhibited a binding energy of −10.2127 kcal/mol, involving residues ASN283, TYR72, and VAL282. In contrast, TGF‐βR (1UHL) displayed a marginally lower affinity of −9.85101 kcal/mol but revealed significant interactions with polar and charged residues such as TYR215, ARG219, and GLU253, potentially influencing downstream signalling modulation [10]. EGFR (7KPB), exhibiting a binding energy of −9.23343 kcal/mol, demonstrated a complex interaction network comprising more than 20 residues, including GLY316, ARG419, and LYS558, indicating a diverse binding mode that may affect epithelial regeneration (Table 2). Finally, PDGFR (2ACL) displayed the least binding affinity at −8.92542 kcal/mol, though it still formed stabilising hydrogen bonds with ASN225, SER54, and ARG81. Collectively, these docking results highlight Trimyristin multi‐target potential in modulating wound healing pathways, with VEGFR and FGFR emerging as particularly responsive targets. For structural insights, each receptor's PDB entry can be accessed via its respective DOI [9].

**TABLE 2:** Docking study for trimyristin phytoconstituent with selected receptor**s**.

Receptor name	PDB ID	Binding energy (kcal/mol)	Docking run time	Key interactions	PDB DOI
EGFR	7KPB	−9.23343	9435 S	316 GLY H‐bond with 315 GLU, 419 ARG H‐bond with 421 ASP, 558 LYS H‐bond with 557 GLU, 532 TYR with 531 HIS, 533 TRP with 539 GLN, 537 LEU, 535 GLU, 534 SER, 366 THR with 365 LEU, 416 with 415 ALA, 414 SER with 416 GLU, 503 ARG with 289 GLU, 500 SER with 519 ASP, 500 SER with 499 CYS, 520 THR with 500 SER, 370 SER with 414 SER, 379 LYS with 378 THR, 380 VAL with 371 ARG, 370 SER with 369 ILE, 416 GLU with 415 ALA, 368 THR with 367 HIS	https://doi.org/10.2210/pdb7KPB/pdb
VEGFR	8HBM	−11.7434	5699 S	H‐bonds with 322 ARG, 330 TYR, 349 GLN, 313 CYS, 319 PHE, 318 GLY, 321 LYS, 323 THR, 328 LEU, 299 ILE, 298 ALA, 341 ASP, 344 GLN, 345 ARG, 352 ARG, 340 ILE	https://doi.org/10.2210/pdb8HBM/pdb
TGF‐βR	1UHL	−9.85101	13576 S	215 TYR, 219 ARG, 230 GLY, 232 SER, 234 ASN, 236 ASP, 253 GLU, 255 LYS, 274 LEU, 275 MET	https://doi.org/10.2210/pdb1UHL/pdb
FGFR	2AZ5	−10.2127	16545 S	H‐bonds with 283 ASN, 72 TYR, 282 VAL	https://doi.org/10.2210/pdb2AZ5/pdb
PDGFR	2ACL	−8.92542	10640 S	H‐bonds with 225 ASN, 54 SER, 81 ARG	https://doi.org/10.2210/pdb2ACL/pdb

### 3.5 Wound Contraction Rates & Epithelialization Time

The Table 3 presents the percentage wound contraction activity observed in rats treated with various gel formulations over a 14 day period, comparing five groups: control, induced, standard, Test I, and Test II. The control group consistently shows 100% wound contraction across all time points, serving as a baseline reference. In contrast, the induced group—representing untreated wound exhibits markedly lower contraction, starting at 9.23% on Day 2 and gradually increasing to 87.39% by Day 14, indicating slower natural healing (Table 3). The standard group, treated with a known therapeutic agent, demonstrates significantly accelerated healing, reaching 37.23% by Day 2 and achieving complete wound closure (100%) by Day 12. Test I and Test II groups, which received experimental gel formulations, show intermediate efficacy in Figure 2. Test II consistently outperforms Test I, with contraction rates of 25.23% versus 17.23% on Day 2, and 93.23% versus 83.45% by Day 10. By Day 12, both treatments reach 100% contraction, suggesting full wound closure, although data for Day 14 is missing for Test II and the standard group. Overall, the results indicate that both experimental treatments enhance wound healing compared to the induced group, with Test II showing performance comparable to the standard therapy.

**TABLE 3:** Percentage wound contraction activity of prepared gel in treated rats.

Days	Control group	Induced group	Standard group	Test I	Test II
Day 2	100	9.23 ± 0.85	37.23 ± 0.34	17.23 ± 0.43	25.23 ± 1.52
Day 4	100	21.34 ± 1.03	53.64 ± 0.75	34.40 ± 1.03	50.34 ± 0.94
Day 6	100	39.45 ± 1.04	78.23 ± 0.77	69.33 ± 1.54	73.23 ± 1.84
Day 8	100	52.99 ± 0.94	89.32 ± 1.04*	78.33 ± 0.78	82.58 ± 1.11*
Day 10	100	67.11 ± 1.12	95.23 ± 2.32*	83.45 ± 1.84*	93.23 ± 1.74*
Day 12	100	73.84 ± 1.34	100	94.69 ± 2.10*	100
Day 14	100	87.39 ± 2.12	—	100	—

Data are represented as mean ± SEM (n = 6), significantly different at *p < 0.05 in comparison to the control group.

**FIGURE 2:**
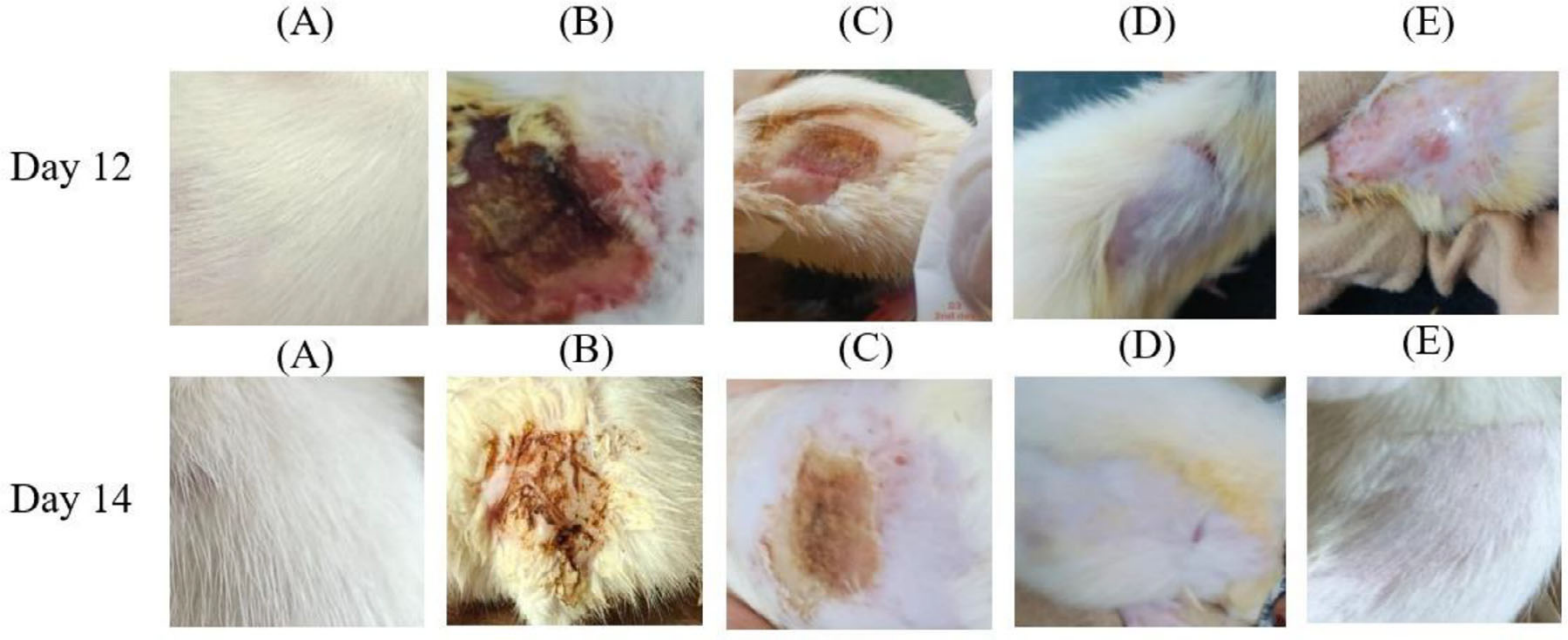
Skin morphology of treated animals after drug treatment application. (A) Control group, (B) Diseased group, (C) Standard group, (D) Treatment group 1, and (E) Treatment group ‐ 2.

#### 3.5.1 Epithelialization Period

The duration of epithelialization period due to the applied nanoemulgel formulations was tested in order to explore their role in acceleration in wound closure in animal models. Burn induction group, without any treatment had an extended period of epithelialization of 14.34 ± 0.84 days which typified physiological duration of epithelialization in untreated burn wounds. On the other hand, the group treated with standard formulation had a much faster healing process than the comparison group, as it needed 10.13 ± 0.77 (*p* < 0.05) days to complete epithelialization, which demonstrated the efficacy of the reference formulation. Of the test formulations, the test II containing higher level of trimyristin (500 mg/mL) exhibited highly superior healing profile with an epithelialization period of 10.95 ± 1.02 days that was comparable to the standard. This indicates that better lipids composition and stronger nanoemulgel properties promoted faster tissue regeneration (Table 4). For Test I, 300 mg/50 mL of trimyristin resulted in a modest curb against the challenged group, but too led to epithelialization in 13.42 ± 0.73 days and the difference between the test and control groups was statistically significant (*p* < 0.05), but less pronounced than Test II.

**TABLE 4:** Effect of topical application of gel on epithelialization period of wounded animals.

Groups	Epithelialization period (days)
Control group	—
Induced group	14.34 ± 0.84
Standard	10.13 ± 0.77*
Test I	13.42 ± 0.73*
Test II	10.95 ± 1.02*

Data are represented as mean ± SEM (n = 6), significantly different at *p < 0.05 in comparison to the control group.

#### 3.5.2 Tensile Strength

The topical application of trimyristin nanoemulgel significantly increased the tensile strength of healed skin in the incision wound model which represents better biomechanical recovery. The 500 mg/50 mL of trimyristin (Test II) showed the highest tensile strength at 412.8 g, suggesting better collagen synthesis and wound dermal matrix arrangement. At least, this value also exceeded the performance of the standard treatment group (398.7 g), and it was concluded that enhanced penetration into and activity of the drug on the skin of the developed enhanced bioavailability system might depend on optimized lipid concentration and the nanoemulgel structure (Figure 3). Even the 300 mg/50 mL form (Test I) showed appreciable improvement to the tensile strength, that is, 362.1 g in the treated induced group upon comparison to the untreated induced group (210.4 g). These findings demonstrated the dose‐dependence effect of trimyristin nanoemulgel on the structural integrity and function of an injured tissue, presumably attributed to enhanced fibroblast activation, deposition of extracellular matrix, and support of angiogenesis.

**FIGURE 3:**
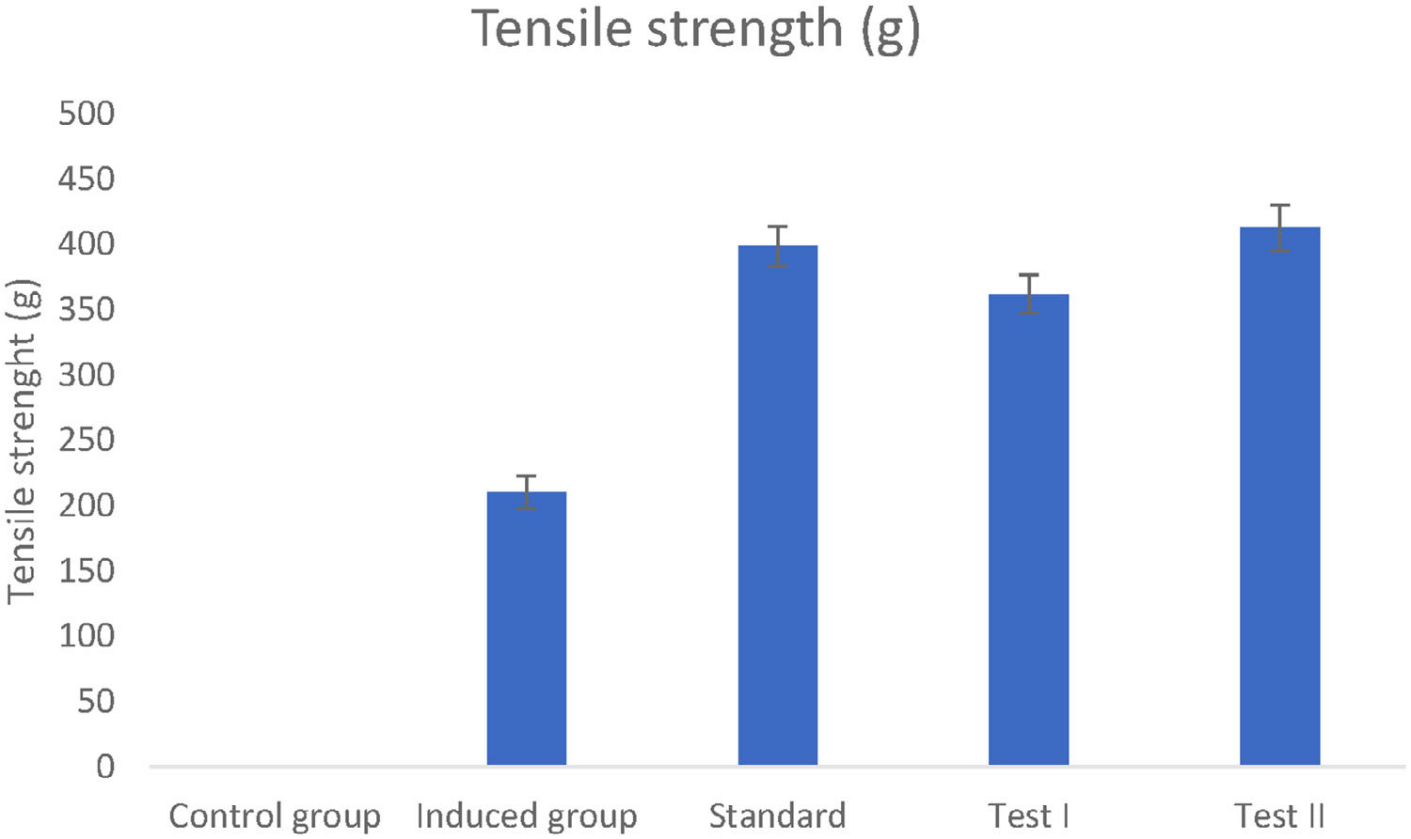
Effect of Trimyristin nanoemulgel on tensile strength of skin fiber.

### 3.6 Histopathological Observations

The histological examination of the control group and the diseased group in the second‐degree burn wound rehabilitation research reveals normal, intact skin architecture. The control group exhibits a healthy skin environment, with intact hair growth follicles and oil glands. The diseased group shows significant tissue damage, including full‐thickness epidermal necrosis and compromised structural integrity. The standard group shows significant enhancement in tissue architecture, with partial re‐epithelialization and moderate organisational structure. Treatment 1 group, administered a low dose of nanoemulsion‐loaded hydrogel, shows optimistic tissue regeneration, suggesting therapeutic efficacy (Figure 4). The treatment 2 group, administered a high dose of the drug‐loaded hydrogel, shows near‐complete tissue regeneration, signifying substantial therapeutic success. The epidermis is well‐structured, with minimal signs of prior injury. The wound environment shifts from inflammation to remodelling, with fully formed skin appendages and restored tissue architecture. These findings suggest that the high‐dose nanoemulsion hydrogel is the most effective treatment group in the current research.

**FIGURE 4:**
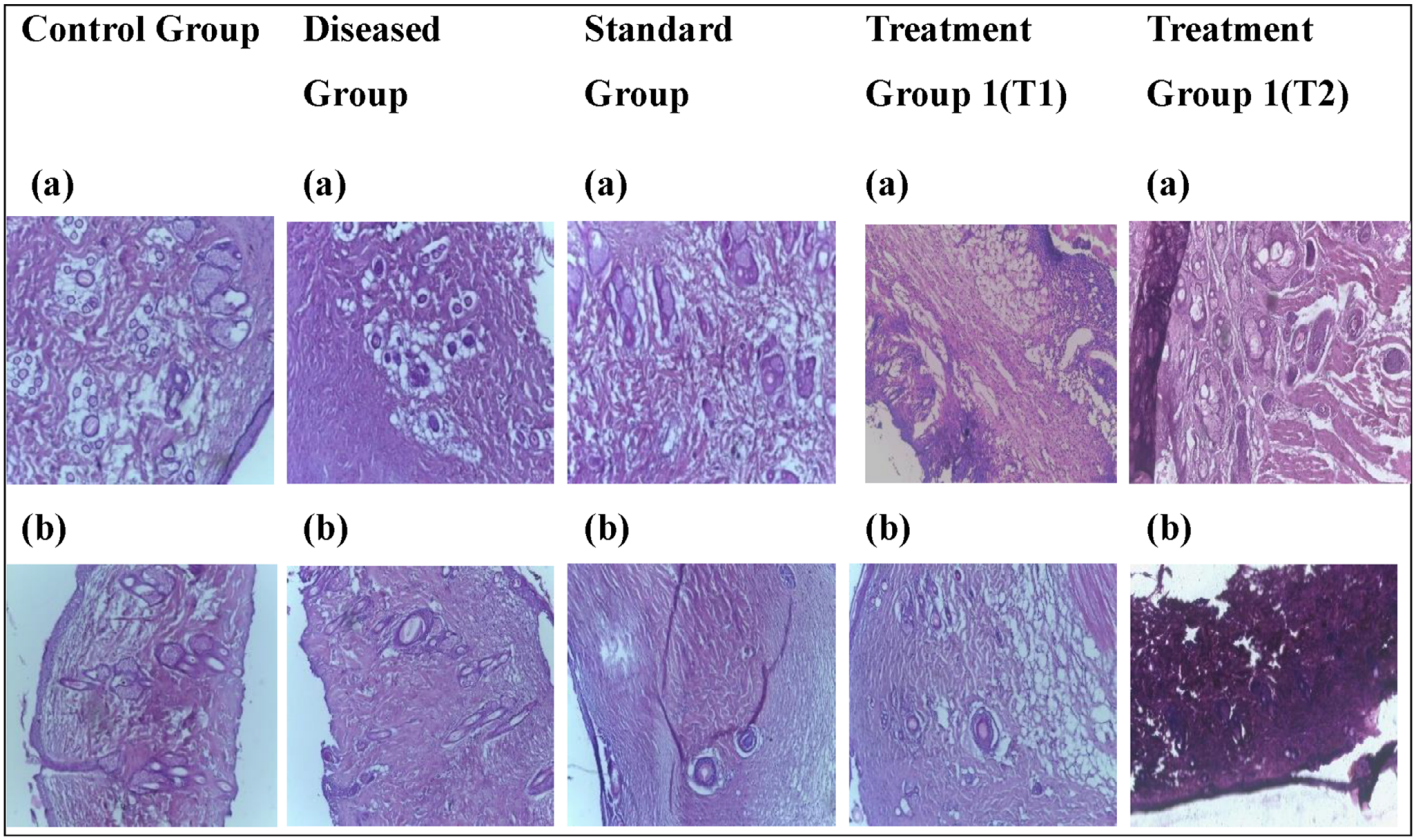
Histopathological analysis of treatment animals.

## 4 Discussion

The current research shows that trimyristin‐loaded nanoemulgel compositions considerably expedite the healing of second‐degree burns, as indicated by increased wound contraction, decreased epithelialization duration, and superior histological results. These findings corroborate and enhance previous studies on lipid‐based nanoemulsion systems for skin healing. Baumann et al. indicated that altering drug lipophilicity in lipid nanoemulsions extended release and enhanced therapeutic retention in inflamed tissues, a principle evident in the sustained release and bioavailability demonstrated in our trimyristin nanoemulgel [30]. The molecular docking results from our investigation demonstrated strong binding affinities of trimyristin to VEGFR and FGFR, suggesting its potential to affect angiogenic and proliferative pathways. This corresponds with the results of Feng et al., who demonstrated that Schizonepeta N annua‐loaded nanoemulsion hydrogels enhanced wound healing via VEGFR‐mediated angiogenesis [31]. Similarly, Dokla et al. developed a benzimidazole‐based nanoemulgel that facilitated re‐epithelialization and collagen deposition. This demonstrates the significance of the nanoemulgel's structure in facilitating skin healing [8]. Our findings confirm the research carried out by Donthi et al., which emphasized the dual advantages of nanoemulgels in improving medication solubility and dermal absorption, particularly for lipophilic compounds [7]. The trimyristin nanoemulgel demonstrates physicochemical characteristics such as suitable viscosity, skin‐compatible pH, and favorable spreadability that conform to the design principles recommended by Prajapati et al. for ocular nanoemulgel systems, suggesting its potential application in mucocutaneous wound conditions [32]. All these results together suggest that trimyristin‐based topical nanoemulgel formulations, especially at the higher concentrations, efficiently support epithelial wound repair and accelerate the wound contracture by probable increment of dermal penetration, anti‐inflammatory effect, and stimulation of angiogenic and fibroblastic pathways.

Histopathological investigation in our work demonstrated almost complete regeneration of tissues in the high‐dose treatment group, consistent with the findings of Morsy et al., which indicated that atorvastatin‐loaded nanoemulgels promoted systematic granulation and decreased inflammation in burn models [26]. Moreover, the zeta potential and particle size distribution identified in our formulation align with the stability criteria established by Shen et al., hence affirming the integrity of our nanoemulsion design. These studies confirm that trimyristin‐based nanoemulgels adhere to recognized therapeutic standards and represent a significant advancement in targeted burn treatment through multi‐receptor interaction and enhanced topical administration [33, 34].

## 5 Conclusion

The present study demonstrates that trimyristin‐loaded nanoemulgel is an effective topical formulation for second‐degree burn wound healing. The optimized nanoemulsion exhibited nanosized droplets (120 ± 10 nm) with a low polydispersity index (0.18 ± 0.02), indicating excellent colloidal uniformity. Molecular docking revealed strong interactions of trimyristin with VEGFR and FGFR, with binding energies of −11.74 and −10.21 kcal/mol, respectively, suggesting modulation of angiogenic and proliferative pathways. In vivo studies showed that the high‐dose nanoemulgel (500 mg/50 mL) achieved complete wound contraction by day 12 and reduced the epithelialization period to 10.95 ± 1.02 days compared with 14.34 ± 0.84 days in the untreated group. Histopathological examination further confirmed near‐complete restoration of skin architecture. Overall, these findings suggest that trimyristin nanoemulgel represents a promising dual‐targeted therapeutic strategy for advanced burn wound management.

## Author Contribution

**Ankita Sinha**: experimental work, writing original draft. **Parag Jain**: conceptualization, supervision. **Alka Patel**: data collection, review writing and editing. **Kushagra Nagori**: resources. **Ajazuddin**: validation.

## Funding

Funding was not received for this work.

## Ethical Statement

Ethical approval for the study was obtained from the Institutional Animal Ethics Committee (IAEC), reference number RCPSR/2024/IAEC/24.

## Conflicts of Interest

The authors declare no conflicts of interest.

## AI Declaration

Chatgpt version 4.0 was used for editing this article.

## Data Availability

The data that support the findings of this study are available from the corresponding author upon reasonable request.
